# Epilysin (matrix metalloproteinase-28) contributes to airway epithelial cell survival

**DOI:** 10.1186/1465-9921-12-144

**Published:** 2011-10-31

**Authors:** Anne M Manicone, Susanna Harju-Baker, Laura K Johnston, Ann J Chen, William C Parks

**Affiliations:** 1 Center for Lung Biology, Division of Pulmonary and Critical Care Medicine, Department of Medicine, University of Washington, Seattle, WA, USA

**Keywords:** Matrix Metalloproteinases, Influenza, Apoptosis, Epithelium

## Abstract

MMP28 is constitutively expressed by epithelial cells in many tissues, including the respiratory epithelium in the lung and keratinocytes in the skin. This constitutive expression suggests that MMP28 may serve a role in epithelial cell homeostasis. In an effort to determine its function in epithelial cell biology, we generated cell lines expressing wild-type or catalytically-inactive mutant MMP28 in two pulmonary epithelial cell lines, A549 and BEAS-2B. We observed that over-expression of MMP28 provided protection against apoptosis induced by either serum-deprivation or treatment with a protein kinase inhibitor, staurosporine. Furthermore, we observed increased caspase-3/7 activity in influenza-infected lungs from *Mmp28^-/- ^*mice compared to wild-type mice, and this activity localized to the airway epithelium but was not associated with a change in viral load. Thus, we have identified a novel role of MMP28 in promoting epithelial cell survival in the lung.

## Background

The epithelium, which forms a continuous barrier to the environment, is a first line defense against pathogens and toxins [1], and this defense function is especially important in lung. In addition to its barrier function, the airway epithelium produces proteins that participate in host defense, and through specialized mucociliary function, performs vital functions in clearance of particles and microbes [2,3].

Key effector proteins in the epithelial responses to injury and infection are the members of the matrix metalloproteinase (MMP) family [4,5]. The epithelium is a source for many MMPs, with most being induced upon challenge. In the respiratory epithelium, induced MMPs have important functions in regulating inflammatory responses and wound repair [6-11]. In contrast to most epithelial MMPs that are not expressed in unperturbed tissue, MMP28 (epilysin) is constitutively produced by the abundant Clara cells of the conducting airways, suggesting that this MMP plays a role in respiratory epithelial cell homeostasis [12].

Studies of macrophage and neuron-derived MMP28 have demonstrated or suggested roles for this proteinase in macrophage migration and neuronal myelination, respectively [12-14]. However, epithelial-derived MMP28 likely performs different functions. In fact, we have found that although respiratory epithelial cells are high expressers of MMP28, its expression is down-regulated in response to acute bacterial infection [12]. This pattern is in contrast to the upregulation of MMP28 we reported for infected macrophages [12].

To better understand the role of MMP28 in epithelial cells, we created stable cell lines over-expressing either wild-type or catalytically-inactive mutant MMP28 in A549 and BEAS-2B cells, two human pulmonary epithelial cell lines. In both cell lines, over-expression of wild-type MMP28 conferred resistance to apoptosis. To examine if this newly-identified *in vitro *function of MMP-28 translated to an *in vivo *phenotype, we tested the *Mmp28^-/- ^*mice for increased susceptibility to epithelial cell apoptosis. Wild-type and *Mmp28^-/- ^*mice were infected with influenza virus, and the whole lung was homogenized for detection of caspase 3/7 activity. Additional samples were immunostained for active caspase 3. We found that *Mmp28^-/- ^*mice had increased caspase 3 activity and this activity localized to the bronchial epithelium. Together, these data support a role for epithelial-derived MMP28 in promoting cell survival.

## Methods

### Generation of Stable Cell Lines

Plasmids containing wild-type or mutant human *Mmp28 *with a V5-HIS tag engineered to the carboxy terminus were provided by Drs. Sara Illman and Jorma Keski-Oja, University of Helsinki. The vector used to generate these constructs was pEF1/V5-His (Invitrogen, Carlsbad, CA) as described [15]. The mutant MMP28 construct contains a single amino acid change (E to A at position 241) within the catalytic domain, rendering it catalytically inactive [15]. These constructs were transfected into A549 (human lung adenocarcinoma cells) and BEAS-2B (transformed bronchial epithelial cells) using lipofectamine (Invitrogen). Cells were then selected with Geneticin (G-418; Invitrogen), and clones expressing MMP28 were detected using immunofluorescent staining for the V5 epitope.

### Immunoblotting and immunofluorescence

Cells were lysed in RIPA buffer [50 mM Tris (pH 8), 150 mM NaCl, 1% NP-40, 0.5% sodium deoxycholate, 0.1% SDS] supplemented with Complete protease inhibitor cocktail (Roche, Indianapolis, IN). After incubation on ice, lysates were cleared of insoluble material by centrifugation and proteins were separated by SDS-PAGE and transferred to Immobilon PVDF membrane (Millipore, Temecula, CA). Membranes were blocked with 5% milk in TBS-T and then probed with anti-V5 HRP (1:2500; Invitrogen). The membranes were then developed by chemiluminescence using SuperSignal West Femto Substrate (Pierce, Rockford, IL).

Immunofluorescence was performed on cells grown on chamber slides and fixed in cold acetone and methanol followed by a 1 hour incubation with FITC-conjugated anti-V5 antibody (Invitrogen). Fluorescence was visualized using an Olympus BX51 microscope.

### Serum-Deprivation

MMP28 over-expressing cells (A549) were allowed to adhere to collagen I coated-chamber slides (BD Biosciences, San Jose, CA) in serum-free media (30,000 cells/well). At serial time-points, images were obtained to enumerate adhered and spread cells. Cells were left in serum-free media for up to 7 days. In separate experiments, cells were allowed to adhere to untreated polystyrene chamber slides in the presence of 10% serum. The following day, the cells were washed in PBS and serum-free media was added to the chambers. Each clone and experiment was performed in duplicate.

### Staurosporine Treatment

Cells were plated at a density of 3 × 10^4 ^cells/well in 96 well format (black plate with clear bottom), in DMEM supplemented with 10% FBS media (Mediatech, Herndon, VA). The next day, the media was removed and replaced with 50 μl of serum-free DMEM with increasing concentrations of Staurosporine (0-2.0 μM), in triplicate (Calbiochem, La Jolla, CA). After 18 hour incubation, an equal volume of lysis buffer plus caspase 3/7 substrate, (z-DEVD)_2 _-R110, was added to each well per manufacture's instructions (Cell Technology, Mountain View, CA). The caspase 3/7 activity was determined at serial times using excitation at 488 nm and emission at 530 nm using a Synergy 4 Hybrid Multi-Mode Microplate Reader (BioTek). In separate experiments, cell viability of staurosporine-treated cells was assessed by incubating the cells with Alamar Blue (Resazurin; Invitrogen), which is reduced by live cells to a fluorescent compound, Resorufin (excitation: 570 nm).

### Influenza Model

A/PR/8/34, a mouse-adapted H1N1 influenza, was originally obtained from ATCC (ATCC# VR-777). Virus was grown in embryonated chicken eggs. Briefly, 100 μL diluted virus stock was injected into a shallow incision in the eggshell. The incision was sealed with melted paraffin and incubated at 37°C for 36-48 hours. The eggs were subsequently incubated at 4°C for greater than 2 hours. Allantoic fluid was isolated from infected eggs and hemagglutinin activity was tested on chicken red blood cells. Virus titers were calculated based on hemagglutinin activity and on MDCK cells according to the method of Reed and Muench [16]. C57BL/6 wild-type mice and *Mmp28^-/- ^*mice were sedated with isofluorane and 100 PFU was delivered via oropharyngeal aspiration in 50 μL sterile PBS. These protocols have been approved by the Institutional Animal Care and Use Committee at the University of Washington.

Three and five days after influenza infection, *Mmp28^-/- ^*and wild-type mice (n = 4/group) were euthanized with Beuthanasia-D (Shering-Plough, Union, NJ) and exsanguination. Uninfected *Mmp28^-/- ^*and wild-type control (n = 4/group) mice were also euthanized at a separate time. In all mice, the left lung was removed and homogenized in 1 ml of PBS. An aliquot was used for caspase 3/7 activity, and the remaining lung homogenate used for RNA isolation with a commercially-available kit (Qiagen, Valencia, CA). The right lung was inflated and embedded in Tissue-Tek O.C.T. medium (Sakura Finetek, Torrance CA), frozen in liquid nitrogen, and cut into 5 μm sections for immunostaining. Additional wildtype and *Mmp28^-/- ^*mice (n = 4-5/genotype/time-point) were infected for collection of whole lung for RNA to assess for MMP expression and viral replication at days 0, 3, 5, and 7. Day 0 mice were harvested immediately after instillation of virus.

### Caspase Activity

For detection of whole lung caspase 3/7 activity, 50 μl of the whole lung homogenate was plated in duplicate on 96 well plates (Costar black plate with clear bottom, Corning Inc.), and an equal volume of lysis buffer plus caspase 3/7 substrate, (z-DEVD)_2 _-R110, was added to each well per manufacture's instructions (Cell Technology). Starting at 15 min, the caspase activity was determined at serial times using excitation at 488 nm and emission at 530 nm using a Synergy 4 Hybrid Multi-Mode Microplate Reader (BioTek).

### Active Caspase 3 Immunostaining

Frozen lung sections (5 μm) were fixed in cold acetone and methanol, washed with PBS and blocked with 5% goat serum for 30 min. The lung was incubated with polyclonal rabbit anti-human cleaved (active) caspase 3 (Biocare Medical, Concord, CA) or rabbit IgG as control, diluted 1:100 in 1% goat serum for 60 min at room temperature, followed by the secondary antibody, goat anti-rabbit IgG conjugated to Alexa 568 (Invitrogen) at a concentration of 1:500. Fluorescence was visualized using an Olympus BX51 microscope.

### RT-PCR: Quantitative Reverse Transcription PCR (qRT-PCR)

Total RNA was isolated from whole lung using an RNeasy Midi kit (Qiagen, Valencia, CA). The quantity and quality of RNA were determined using a NanoDrop spectrophotometer (NanoDrop Inc., Wilmington DE). cDNA was synthesized from 5 μg total RNA with a High-Capacity cDNA Archive kit (Applied Biosystems), and then treated with RNaseH. Primers and probes (FAM dye-labeled) for HPRT and influenza (5' primer CATCCTGTTGTATATGAGGCCCAT; 3' primer GGACTGCAGCGTAGACGCTT) were added, and product amplification was measured with an ABI HT7900 Fast Real-Time PCR System. The threshold cycle (Ct) was obtained from duplicate samples and averaged. The ΔCt was the difference between the average Ct for influenza and HPRT. The ΔΔCt was the ΔCt of the *Mmp28^-/- ^*samples minus the average ΔCt of matched wild-type samples. The data are expressed as relative quantification (RQ), which is the fold change and calculated as 2^-ΔΔCt^.

### Statistics

Results are expressed as means ± SEM. Statistic significance was determined using the Student's *t *test. Differences were considered significant if the p value was < 0.05.

## Results

### Generation of Stable Cell Lines

**Figure 1:**
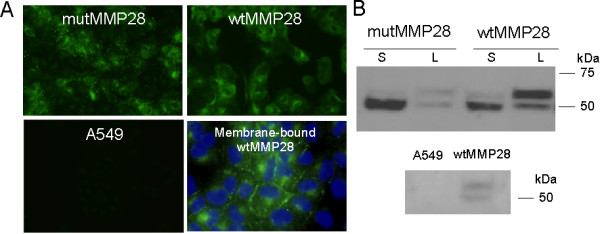
**Representative images demonstrating expression of recombinant, V5-tagged wild-type and mutant MMP28 in A549 cells**. A. Immunofluorescence (IF) using FITC-conjugated anti-V5 antibody. Bottom right image demonstrates localization of MMP28 to the plasma membrane. B. Western blot (WB) of supernatants (S) and cell lysates (L) from representative mutant and wild-type clones. Membranes were stained with anti-V5 HRP. The larger band (58 kDa) represents the pro-form, which is more abundant in the cell lysate, and the smaller band (48 kDa) represents the secreted active form, which is more abundant in the supernatant. A549 cells had no staining by IF or WB using the V5 antibodies.

To establish stable cell lines over-expressing wild-type or mutant MMP28, we transfected A549 and BEAS-2B cells with plasmids containing full-length wild-type (wtMMP28-V5) or catalytically-inactive (mutMMP28-V5) MMP28. Each construct was tagged with a V5 epitope on the carboxy terminus, and the mutant construct was engineered with a single amino-acid change within the catalytic-domain, rendering it inactive as previously reported [15]. Transfected cells were selected for antibiotic resistance with Geneticin, and two wild-type clones and two mutant clones were identified as expressers of MMP28 using immunostaining with commercially-available V5 antibodies (Figure 1A). We also identified cells that expressed no recombinant MMP28 (A549 neg) but were maintained under the same culture conditions (data not shown).

In both cell lysates and supernatant from the mutant and wild-type clones, we identified both the zymogen (proMMP28; 58 kD) and the furin-cleaved (MMP28; 48 kD) forms of the enzyme (Figure 1B). As expected, most of the protein in the supernatant was the 48-kD activated enzyme (of course, the E-A mutant form does not gain activity after the prodomain is cleaved). In addition to its diffuse cytoplasmic and peri-Golgi staining, MMP28 localized to the cell membrane as described by others [15]. However, in contrast to previous findings [15], we found no change in the morphology of cells expressing wild-type (or mutant) MMP28. Furthermore, the ability of cells to adhere to type I collagen or to close scrape wounds [17] was not influenced by expression of wildtype or mutant MMP28 (data not shown).

### MMP-28 Promotes Cell Survival

**Figure 2:**
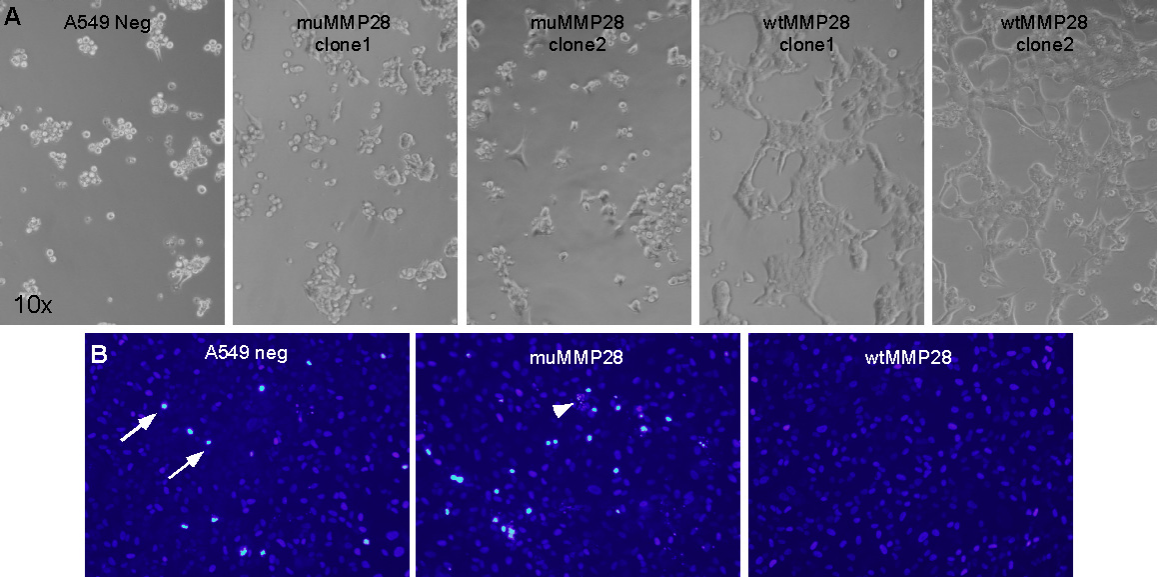
**Over-expression of wild-type MMP28 confers a cell-survival advantage during serum-deprivation conditions**. An equal number of A549 neg, wtMMP28 and muMMP28 cells (1-2 clones each, performed in duplicate) were plated onto collagen I-coated chamber slides in the absence of serum. (A) After 6 days in culture, the above images were obtained demonstrated floating cellular debris in the A549 neg and mutant clones (left 3 columns) and many viable cells in the wild-type clones (right 2 columns). Notably, initial adherence and spreading of the cells on collagen were similar, but survival after six-days of serum-deprivation was enhanced in clones expressing active MMP28. (B) Assessment of nuclear condensation and fragmentation (associated with apoptosis) using DAPI staining of serum-deprived cells at 24 h, revealed increased condensed (arrows) and fragmented (arrowhead) nuclei in only the A549 neg, muMMP28 cells and not wtMMP28.

**Figure 3:**
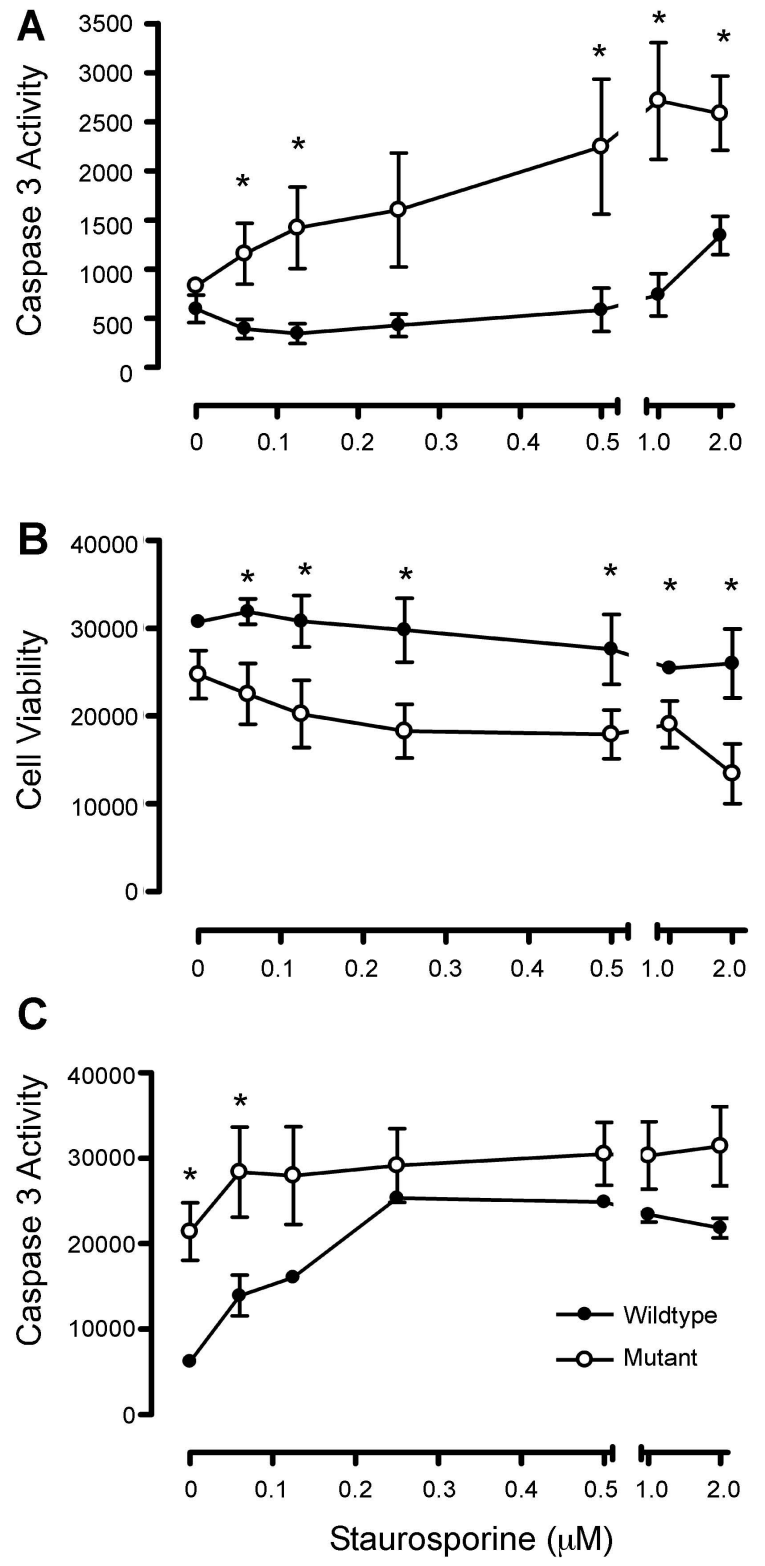
**Expression of wild-type MMP-28 protects against staurosporine-induced apoptosis in A549 and BEAS-2B cells**. A549 (A, B) or BEAS-2B (C) cells expressing wild-type MMP-28 (closed circle) or mutant MMP-28 (open circle) were plated on a 96-well plate with increasing doses of staurosporine in serum-free medium (0 - 2 μM). After 16 h incubation with staurosporine or serum-free medium, the level of caspase 3/7 activity was determined by measuring the fluorescence of a cleaved caspase 3/7 substrate (A,C). In separate experiments, the cell viability was assessed by Alamar Blue staining (B).

We observed that A549 cells expressing wild-type MMP28 had increased survival rates during serum-deprivation compared to cells expressing mutant or no MMP28. After 6 days in serum-free medium, only wild-type clones remained viable (Figure 2A). Assessment of nuclear condensation, one morphologic change associated with apoptosis, using DAPI staining at 24 h after serum deprivation, demonstrated increased condensed nuclei in A549 neg and muMMP28 cells when compared to wtMMP28 cells (Figure 2B). To explore these observations further, we induced apoptosis in cells with increasing doses of staurosporine and assessed caspase 3/7 activity 16 h later. At all doses of staurosporine, we observed less caspase 3/7 activity in cells expressing wtMMP28-V5 compared to cells expressing catalytically inactive MMP28 (Figure 3A). These findings were confirmed in other experiments in which cells were treated with increasing doses of staurosporine for 16 h and cell viability was measured with Alamar Blue (Figure 3B). A549 neg cells, expressing no recombinant MMP28 protein, behaved similar to muMMP28 clones (data not shown). Together, these results indicate that active MMP28 promotes epithelial cell survival.

To assess if this survival effect was mediated in other epithelial cells, we examined if MMP28 could confer resistance to staurosporine-induced apoptosis in BEAS-2B cells, a human pulmonary epithelial cell line. Stable cell lines were created using wild-type and mutant human MMP28, and two positive clones of each were assessed for their ability to resist apoptosis using a caspase 3/7 activity assay. As seen with A549 cells, we observed less staurosporine-induced caspase 3 activity in BEAS-2B cells expressing wtMMP28-V5 compared to mutant-expressing cells (Figure 3C). However, this protective effect of wild-type MMP28 was seen at the lowest doses of staurosporine (0.062 and 0.125 μM). At higher doses, BEAS-2B cells were overall more sensitive to staurosporine-induced apoptosis than were A549 cells. We also observed increased caspase 3/7 activity in the mutant cells in the no staurosporine (0 μM) group, likely reflecting differences in apoptosis induced by serum-deprivation.

### *Mmp28*^-/- ^Mice have increased airway epithelial cell apoptosis

**Figure 4:**
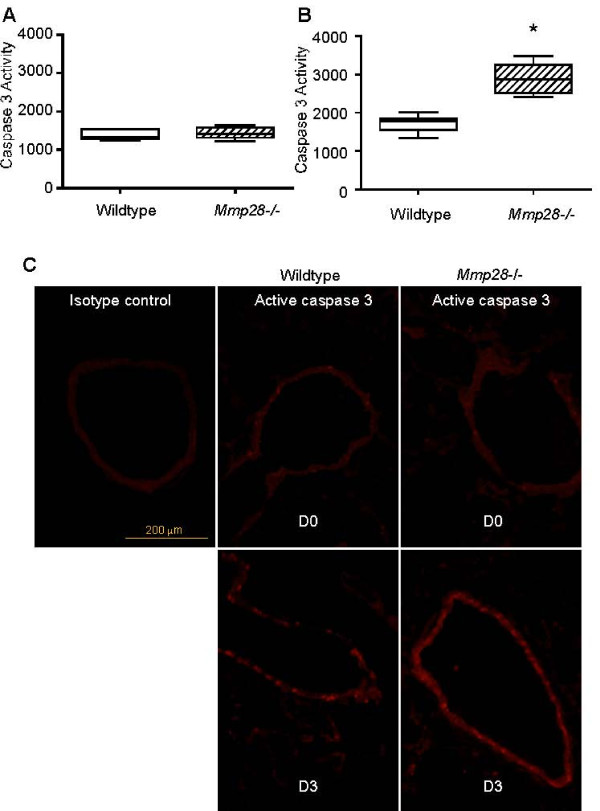
**Increased Caspase 3 Activity in the airway epithelium of influenza infected *Mmp28^-/- ^*Mice**. C57BL/6 wild-type mice and *Mmp28^-/- ^*mice received 100 PFU of mouse-adapted H1N1 influenza (A/PR/8/34) via oropharyngeal aspiration. At days 3 and 5 post-infection, the left lung was homogenized for caspase 3/7 activity, and the right lung was processed for immunohistochemistry using rabbit anti-cleaved (active) caspase 3 antibody, and anti-rabbit IgG conjugated to Alexa 568. (A) At day 0, there was similar background caspase 3/7 activity in the lungs from wild-type and *Mmp28-/- *mice. (B) At day 3, there was increased caspase 3/7 activity in *Mmp28^-/- ^*lungs, and (C) this activity localized to the bronchial epithelium at day 3. There was no significant caspase staining in the airways at day 0 in either genotype. (n = 4-5 mice/genotype/timepoint). *p value < 0.05

To examine if MMP28 influences cell survival in vivo, we tested the *Mmp28^-/- ^*mice for increased susceptibility to airway epithelial apoptosis. Because influenza infection targets the airway epithelium and leads to epithelial cell apoptosis [18-20], we assessed if influenza infection led to increased apoptosis in *Mmp28^-/- ^*epithelium. Age and sex matched *Mmp28^-/- ^*and wild-type mice were infected with murine influenza strain A/PR/8/34 at 100 PFU/mouse. On day 3 and 5 post-infection, caspase 3/7 activity was measured in homogenates of the left lungs. We found that *Mmp28^-/- ^*mice had a significant increase (68.3 ± 7.65%; p < 0.0006) in whole lung caspase 3/7 activity compared to the level detected in wild-type homogenates at day 3 (Figure 4B) but not at day 5 (not shown). In the same experiment, the right lung was collected and processed for immunostaining with an anti-active caspase 3 antibody. In both wild-type and *Mmp28^-/- ^*mice, most (>95%) caspase 3 activity localized to the airway epithelium at day 3 (Figure 4B). At day 0 (uninfected), there was no difference in caspase 3/7 activity between genotypes and there was markedly less active caspase 3 staining in the airway compared to day 3 (Figure 4A, C).

**Figure 5:**
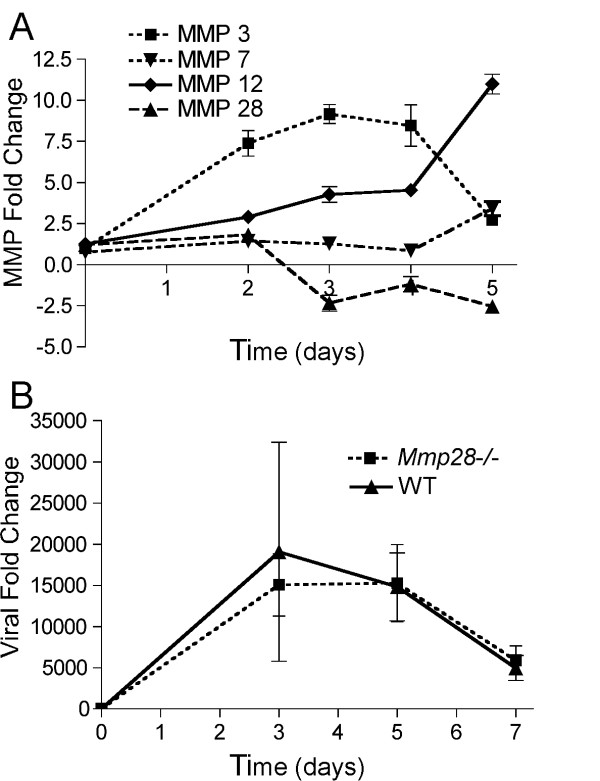
**MMP28 expression in the lung decreases in influenza infection and does not contribute to viral replication or clearance**. C57BL/6 wild-type and *Mmp28^-/- ^*mice were infected with 100 PFU of mouse-adapted H1N1 influenza (A/PR/8/34), and the lungs were harvested at serial time-points for RNA isolation. (A) Expression of MMPs -3,-7,-12, and -28 was assessed using real-time PCR. Only MMP28, which is constitutively expressed in the naïve lung, decreased during infection. (n = 4 mice/time-point). P value <0.05 for all MMPs when compared to their respective baseline expression at D0, except for MMP7 at D4. (B) Viral titers as assessed by RT-PCR from lung homogenates did not differ between wild-type and *Mmp28^-/- ^*mice (n = 4-5 mice of each genotype/time-point).

Since airway epithelial apoptosis has been proposed as a mechanism for viral clearance [21], we hypothesized that increased apoptosis in the *Mmp28^-/- ^*mice would result in faster viral clearance. Viral levels were assessed by using real-time PCR of the viral genome sequences at days 0, 3, 5, and 7 days post-infection. Despite differences in epithelial cell apoptosis at 3 days, we found that influenza virus levels were similar at all time-points in *Mmp28^-/- ^*and wild-type mice, suggesting that MMP28 is not required for control of influenza infection (Figure 5B). Furthermore, we found that MMP28 mRNA expression from influenza-challenged whole lung was unchanged in early infection (days 1-2) and decreased at later timepoints (days 3-5), in contrast to other MMPs -3,-7,-12 (Figure 5A). This down-regulation of MMP28 by epithelial cells has been seen in other pulmonary infection models[12], and may be one mechanism by which host epithelial cells respond to infection. Furthermore, this delay in MMP28 down-regulation may explain our findings of differences in caspase 3/7 activity at day 3 but not day 5.

## Discussion

The respiratory epithelium in the lung has adapted to continuous environmental exposures and pathogenic stimuli. It serves to protect the host from organisms through barrier function, mechanical clearance, secretion of inhibitors of bacterial growth, and release of chemokines and other factors to recruit and activate leukocytes. Given the constant environmental exposures challenging the epithelium, mechanisms have evolved to restrain inflammatory responses and promote epithelial survival. Our studies suggest that constitutive expression of epithelial MMP28 contributes to this latter process.

Our studies demonstrate a novel role for MMP28 in promoting epithelial cell survival. Both in vitro and in vivo studies indicate that MMP28 promotes airway epithelial cell survival when challenged by virus, serum-deprivation, or staurosporine. These studies provide some insight as to potential evolutionary pressures that have resulted in epithelial cell down-regulation of MMP28 during infection as a potential mechanism to facilitate apoptosis of injured or infected epithelium.

Although other groups have reported that influenza infection is a potent inducer of epithelial cell apoptosis[18-20], one limitation of our in vivo studies is that caspase 3/7 activity may also regulate non-apoptotic pathways. Another limitation of our study is that A549 cells, which are tumor-derived alveolar epithelial cells, and BEAS-2B cells, which are immortalized human bronchial epithelial cells, have altered survival and apoptotic pathways that differ from normal. Hence, our findings will need to be confirmed in primary epithelial cells as well. However, these studies highlight another potential role for MMP28 in cancer cell biology, as MMP28 expression has been associated with several malignancies [22,23]. In our studies of *Mmp28^-/- ^*and wild-type mice crossed with *K-ras ^LA1 ^*mutant, we found a significant decrease in spontaneous lung tumor formation in the *Mmp28^-/- ^*mice compared to wild-type mice (unpublished findings). Although we have not yet linked these findings to differences to apoptosis, potentially, the presence of MMP28 may confer a survival advantage leading to more lung tumors in this model.

The mechanism by which MMP28 contributes to cell survival remains unknown. Since MMP28 localizes to the cell membrane, it is likely that its substrate resides in close proximity and may be a receptor or other membrane-associated protein that participates in cell survival pathways. Such factors include growth factors (EGF/IGF-1/TGFα) and their receptors which signal through PI3K, Ras or JAK-STAT pathways to upregulate gene transcription of factors that promote cell survival [24].

Other metalloproteases function in regulating apoptotic and anti-apoptotic pathways. For example, TACE/ADAM17 is responsible for shedding of the pro-tumor necrosis factor (proTNF-α) [25]. Furthermore, it is also necessary for shedding of the EGF family of growth factors [26], which function in cell survival pathways. MMP7 mediates apoptosis via cleavage of FasL, making cells more resistant to apoptosis [27]. Cleavage of death domain receptors (such as FAS) or inactivation of their ligands could interfere with these specific apoptotic pathways, but are unlikely mechanisms in MMP28 mediated cell survival since the challenges used to induce apoptosis (serum deprivation, staurosporine and viral infection) do not share these pathways; however, roles of MMP28 in shedding of growth factors remains unknown.

Interestingly, we did not observe any features of an epithelial-to-mesenchymal transformation (EMT), such as loss of cell-cell contacts or accelerated wound closure or migration, in MMP28 over-expressing cell lines, as reported by others [15]. However, resistance to apoptosis is a property of EMT [28-30], and we may have observed an "intermediate" phenotype in our studies. Differences between our phenotypes and those reported by others may relate to the relative abundance of MMP28 expression due to promoter and vector differences, or differences in the cell lines[31]. However, since MMP28 is constitutively expressed in epithelial cells, it is unlikely that this proteinase participates in cellular transformation in vivo. We propose that MMP28 functions normally to resist apoptosis, and indeed, the enhanced cell death we observed in virally infected *Mmp28^-/- ^*mice supports this conclusion. Ongoing studies are aimed at determining the mechanism by which MMP28 promotes cell survival.

## Conclusion

MMP28 is constitutively expressed by the respiratory epithelium, suggesting roles in maintaining homeostasis. We have found that expression of catalytically-active MMP28 in lung epithelial cells promotes cell survival. Furthermore, *Mmp28-/- *mice demonstrate increased susceptibility to influenza-induced respiratory epithelial cell apoptosis. These studies identify a previously unknown role for MMP28 in respiratory cell biology.

## Competing interests

The authors declare that they have no competing interests.

## Authors' contributions

SHB assisted with stable cell line generation, AJC and LKJ assisted with influenza infections, RT-PCR and IHC studies, AMM performed caspase activity assays, designed experiments, wrote manuscript, WCP contributed to design and manuscript review. All authors read and approved the final manuscript.
